# Growth and puberty in children with juvenile idiopathic arthritis

**DOI:** 10.1186/s12969-021-00521-5

**Published:** 2021-03-12

**Authors:** Debora Mariarita d’Angelo, Giulia Di Donato, Luciana Breda, Francesco Chiarelli

**Affiliations:** Department of Pediatrics, University of Chieti, Chieti, Italy

**Keywords:** Juvenile idiopathic arthritis, Puberty, Growth, Bone, Hormone replacement therapy

## Abstract

Juvenile Idiopathic Arthritis is one of the most prevalent chronic diseases in children, with an annual incidence of 2–20 cases per 100,000 and a prevalence of 16–150 per 100,000. It is associated with several complications that can cause short-term or long-term disability and reduce the quality of life. Among these, growth and pubertal disorders play an important role. Chronic inflammatory conditions are often associated with growth failure ranging from slight decrease in height velocity to severe forms of short stature. The prevalence of short stature in JIA varies from 10.4% in children with polyarticular disease to 41% of patients with the systemic form, while oligoarthritis is mostly associated with localized excessive bone growth of the affected limb, leading to limb dissymmetry. The pathogenesis of growth disorders is multifactorial and includes the role of chronic inflammation, long-term use of corticosteroids, undernutrition, altered body composition, delay of pubertal onset or slow pubertal progression. These factors can exert a systemic effect on the GH/IGF-1 axis and on the GnRH-gonadotropin-gonadic axis, or a local influence on the growth plate homeostasis and function**.** Although new therapeutic options are available to control inflammation, there are still 10–20% of patients with severe forms of the disease who show continuous growth impairment, ending in a short final stature. Moreover, delayed puberty is associated with a reduction in the peak bone mass with the possibility of concomitant or future bone fragility. Monitoring of puberty and bone health is essential for a complete health assessment of adolescents with JIA. In these patients, an assessment of the pubertal stage every 6 months from the age of 9 years is recommended. Also, linear growth should be always evaluated considering the patient’s bone age. The impact of rhGH therapy in children with JIA is still unclear, but it has been shown that if rhGH is added at high dose in a low-inflammatory condition, post steroids and on biologic therapy, it is able to favor a prepubertal growth acceleration, comparable with the catch-up growth response in GH-deficient patients**.** Here we provide a comprehensive review of the pathogenesis of puberty and growth disorders in children with JIA, which can help the pediatrician to properly and timely assess the presence of growth and pubertal disorders in JIA patients.

## Background

Juvenile Idiopathic Arthritis (JIA) is one of the most prevalent chronic diseases in children. It encompasses a heterogeneous group of arthritis of unknown origin beginning before the 16th birthday and lasting more than 6 weeks [1]. It is estimated that JIA has an annual incidence of 2–20 cases per 100,000 and a prevalence of 16–150 per 100,000 in high-income countries [2]. The etiology is still not completely elucidated, but it seems to be linked to a combination of genetic and environmental factors [1]. According to the International League of Associations for Rheumatology Classification (ILAR), seven subtypes of JIA have been identified, which differ for age and modality of presentation, clinical signs and symptoms, genetic background and prognosis: systemic JIA (sJIA), oligoarthritis, rheumatoid factor (RF)-positive and negative polyarthritis, psoriasic arthritis (PsA), enthesitis-related arthritis and undifferentiated arthritis [3]. Each subgroup of disease is associated with various degrees of severity and complications that can cause short-term or long-term disability and reduce the quality of life. Among these, growth and pubertal disorders play an important role. Thus, the aim of this paper is to supply a full review of the growth and pubertal disorders associated with JIA and to guide the pediatric rheumatologists and the general physicians in the follow up of these patients, in order to timely identify and properly treat children with growth and pubertal alterations.

## Growth and JIA

Chronic inflammatory conditions are often associated with growth failure ranging from slight decrease in height velocity to severe forms of short stature. The prevalence of short stature varies from 10.4% in children with polyarticular disease to 41% of patients with the systemic form [4]. Oligoarthritis is mostly associated with localized excessive bone growth of the affected limb, with premature fusion of epiphyses leading to limb shortening in the older child; early treatment with intra-articular glucocorticoids (GCs) helps to prevent the onset of limb dissymmetry [5]. Data from the Childhood Arthritis Prospective Study (CAPS) showed that a cohort of children with JIA experienced growth restriction over the first 3 years of disease. Although height was within the normal range at presentation, growth restriction, defined as change of height z-score less than 0.5, was observed in 39% of patients and occurred early at disease onset, being greater in patients with sJIA and PsA. Forty-one per-cent of patients with persistent oligoarthritis experienced moderate growth restriction, more important in children who underwent several intra-articular corticosteroid injections. Higher childhood health assessment questionnaire (CHAQ) scores, longer duration of symptoms and lower Body Mass Index (BMI) z-score, were significantly associated with lower height z-scores, like was total time on oral or intravenous steroid treatment with decrease in height z-score from baseline to 3 years. Surprisingly, lower height at baseline predicted improvement of vertical growth over the first 3 years of follow up [6]. Some authors used data from the Research in Arthritis in Canadian Children emphasizing Outcomes (ReACCh-Out) cohort to assess growth in 1147 children newly diagnosed with JIA from 2005 to 2010. They reported that children with the most frequent JIA categories (oligoarthritis, RF-negative polyarthritis, enthesitis-related arthritis and undifferentiated arthritis) grew and gained weight as healthy children, while about 10% of children with sJIA, uncontrolled disease and/or prolonged use of steroids had an increased risk of growth impairment. A minor, but measurable, risk of growth failure was seen in children with RF-positive polyarthritis and PsA [7].

### Systemic and local regulation of linear growth

Linear bone growth occurs at the growth plate and in consequent to a process of chondrogenesis and remodeling of cartilage into bone tissue (a process named “endochondral ossification”). The growth hormone (GH) and the insulin-like growth factor 1 (IGF-1) are important stimulators of longitudinal bone growth. According to the “somatomedin hypothesis”, GH exerts its activity through the liver-derived IGF-1, which regulates proliferation, differentiation and apoptosis of many cell types [8]. GH binds to two receptors (GHRs) on the cell surface, causing a dimerization process and activating a series of other signalling molecules, like phosphatidyl-inositol-3-kinase (PI3K), mitogen-activated protein kinases (MAPKs), insulin receptor substrates (IRSs), protein kinase C (PKC), signal transducer and activator of transcription (STAT) etc. The GH binding protein (GHBP) is proteolyzed from the cell surface receptor and regulates GH bioavailability. GH also acts directly at the growth plate level to recruit resting chondrocytes into the proliferative state, with a subsequent increase local IGF-1 production, which stimulates chondrocytes proliferation and hypertrophy with an autocrine/paracrine mechanism (“dual effector hypothesis”). At a systemic level, six IGF binding proteins (IGFBPs), Acid Labile Subunit (ALS) and the mannose-6-phosphate receptor (type 2 IGF-1 receptor) are the main regulators of IGF-1 bioavailability. In fact, many studies showed that the most important regulator of growth is the level of free IGF-1 (not just the total IGF-1), which is also influenced by its binding to regulatory proteins, above all the IGFBP-3 [9].

## Pathogenesis of growth disorders in JIA

The pathogenesis of growth disorders is multifactorial and includes the role of chronic inflammation, long-term use of supra-physiological doses of corticosteroids, undernutrition, altered body composition with lean mass reduction, physical inactivity, delays of pubertal onset or slow pubertal progression [10]. Also, the degree, extent and duration of disease activity are important, like the age at onset of the disease. These factors can exert a systemic effect on GH-IGF-1 axis, or a local influence on the growth plate homeostasis and function [11]. Growth in children with JIA is also influenced by sex steroids, thyroid hormone, parathyroid hormone-related peptide, bone morphogenic proteins, transforming growth factors and vitamin D metabolites.

## Growth disorders in JIA and inflammation

Inflammation is characterized by the activation of different immune cells with the production of cytokines, chemokines, interleukins (ILs) and interferons (IFN), activating bone reabsorption and inhibiting bone formation at a systemic and/or local level. GH and IGFs are the most important regulators of linear growth. Chronic inflammation can interfere with the function of GH-IGF-1 axis by inducing relative GH insufficiency, GH/IGF-1 resistance, downregulation of GH/IGF-1 receptors, dysregulation of IGFBPs and thus of IGF-1 bioavailability, impairment of local GH and IGF-1 signaling pathways and gene regulation [12]. Several studies demonstrated the role of pro-inflammatory cytokines in influencing growth in children with chronic diseases and in animal models: interleukin-6 (IL-6), interleukin-1β (IL-1β) and tumor necrosis factor-α (TNFα) have been reported to have a major role in JIA.

### JIA inflammatory cytokines: systemic effects on the GH-IGF1 axis

Elevated levels of IL-6 are found in serum and in synovial fluid of patients with JIA and they are associated with disease activity and laboratory markers of severity [13]. These patients, have reduced levels of circulating IGF-1, with normal GH. De Benedetti et al. [14] studied the effects of IL-6 on growth regulation using the neuro-specific enolase (NSE)/hIL-6 transgenic murine model, that overexpresses IL-6: NSE/hIL-6 mice have normal liver IGF-1 production, reduced levels of 3 IGFBP-3 and increased IGFBP-3 proteolysis, resulting in a marked decrease of circulating 150-kDa ternary complex, even in the presence of functional ALS. Low concentrations of IGFBP-3 are also found in sJIA, with a marked decrease in IGF-1 half-life and increased IGF-1 clearance. IGFBP-3 is produced by Kupffer cells, which express IL-6 receptor, thus IL-6 may directly affect IGFBP-3 production or increase its proteolysis through the stimulation of proteases secretion (catepsin B and L, metalloproteinases MMP) from various cell types [11]. Furthermore, IL-6 and other pro-inflammatory cytokines induce the suppressor of cytokine signaling (SOCS) proteins, which reduce and regulate GH signaling; this may explain the GH resistance observed in inflammatory diseases [15]. Pro-inflammatory cytokines may also alter growth plate function by inhibiting IGF-1 intracellular signaling. TNF-α, IL-1β and IL-6 attenuate IGF-1-induced activation of both MAPK and PI3K pathways in chondrocytes [16]. In addition, TNF-α and IL-1β inhibit the IGF-1-mediated phosphorylation of insulin receptor substrate-1 (IRS-1) in myoblasts, but it is unknown if this also occurs in chondrocytes [17]. Finally, IL-1β over-expression reduces IGF-1 and ALS plasma levels. In septic rats, hepatic expression of IGFBP-1 is increased, which reduces IGF-1 bioavailability: this effect is completely reversed by treatment with an IL-1 receptor antagonist [18].

### JIA inflammatory cytokines: local effects on the growth plate

**Fig. 1:**
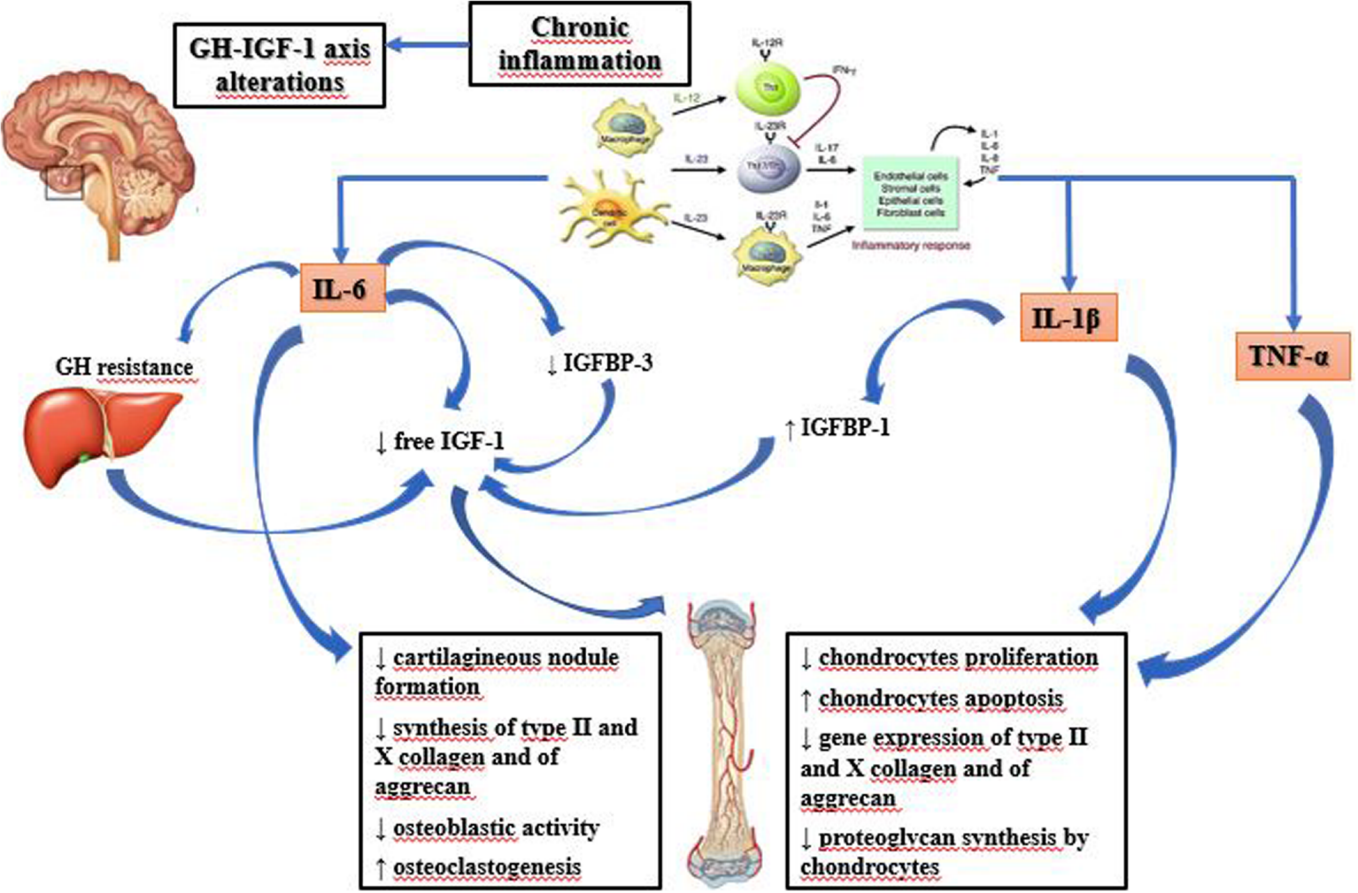
Effects of pro- inflammatory cytokines on GH-IGF1 axis. GH (Growth Hormone); IGF-1 (Insulin-like Growth Factor-1); IGFBP-1 (Insulin-like Growth Factor Binding Protein-1); IGFBP-3 (Insulin-like Growth Factor Binding Protein-3); IL-6 (Interleukin-6); IL-1β (Interleukin-1β); TNFα (Tumor Necrosis Factor-α)

In addition to its systemic effects, IL-6 also acts on the growth plate environment and function reducing cartilagineous nodule formation, with an effect on the early differentiation steps of chondrocytes precursors [19]; it also decreases the synthesis of type II and X collagen and aggrecan. IL-6 can activate janus kinase (JAK)/ STAT signaling leading to down-regulation of type II collagen, aggrecan and link protein transcription in the articular chondrocytes [20]. In vivo studies also showed that IL-6 over-expression increases osteoclastogenesis with augmented bone reabsorption and osteopenia, reduces osteoblastic activity, determines defective ossification and decreases mineral apposition rate [21]. JIA is characterized by important changes in the articular environment and the immune cell proliferation causes localized hypoxia and pH reduction, thus influencing osteoblast function and bone mineralization [22]. Cartilage oligomeric matrix protein (COMP) is produced by chondrocytes and synovial cells and it is a marker of chondrocytes turnover in the growth plate. Children with sJIA have decreased COMP serum levels during active phases of disease, and those levels significantly increase after treatment with the anti-IL-6 tocilizumab. Likewise, serum bone alkaline phosphatase (BAP) concentration, which is a marker of osteoblast activity, is reduced during active disease and increased during remission phases. The active inflammatory state also increases MMP-3 concentration, which is useful for predicting joint damage [23]. While IL-6 acts mainly via systemic mechanisms on GH function, other cytokines, like IL-1β and TNF-α, act primarily on the growth plate with an additive effect. They decrease the width of the proliferating zone of growth cartilage and the rate of endochondral ossification, reducing chondrocytes proliferation and stimulating apoptosis of proliferative cells. A possible mechanism of the induction of apoptosis is the down-regulation of gene expression of Sox-9, a regulatory factor for chondrocyte differentiation and cartilage formation [24]. Some authors found that the exposure of murine ATDC5 chondrogenic cells and metatarsals to IL-1β and TNF-α caused a decreased expression of collagen types II and X and aggrecan [19]. This study also showed a restricted potential for recovery of chondrogenesis and longitudinal bone growth after prolonged exposure to pro-inflammatory cytokines, thus explaining greater growth impairment in children with longer periods of symptoms before diagnosis and appropriate therapy initiation [10]. The most important effects of pro-inflammatory cytokines on GH-IGF-1 axis and on the growth plate are summarised in Fig. 1.

## Growth disorders in JIA and therapy

Patient with JIA, especially those with systemic disease, often need to be treated with systemic steroids for long periods. GCs can inhibit GH pulsatile release, reduce GH and IGF-1 receptors expression by chondrocytes and impair IGF-1 signaling at the growth plate, mainly via the PI3K pathway [25]. GCs also reduce chondrocyte proliferation and differentiation by disrupting cell cycle progression and promoting cell cycle exit [26]. Finally, they stimulate chondrocytes’ autophagy and apoptosis, by altering the amount of pro-apoptotic members of the Bcl-2 family like Bax and Bid [27]. The decreased cell proliferation during GCs treatment preserves chondrocytes proliferative capacity; therefore, after GCs discontinuation, growth plate chondrocytes are less senescent and have a greater proliferative potential [26]. That is why, after remission, growth often accelerates beyond the normal growth rate for age, a phenomenon called “catch up growth” and observed in many growth-retarding conditions, like Cushing’s syndrome, celiac disease and hypothyroidism. However, catch up growth in children may not be complete after discontinuation of GC if inflammation is ongoing [10]. Skeletal development and growth are reversibly impaired during GC treatment, but significant short stature or deviation from the target height has been reported only in children treated with systemic steroids for more than 1 year [28]. Moreover, it is difficult to differentiate the impact of GC therapy and of uncontrolled inflammation on growth impairment, even because the severity of the disease often correlates with the likelihood of treatment with GCs. Steroid treatment has a double role: it can delay or improve growth in patients with JIA, since its anti-inflammatory effect may be positive especially in patients with sJIA, in whom active inflammation and high IL-6 levels may potentially affect growth [29]. Simon D. et al. retrospectively analyzed linear growth patterns during and after GC therapy in 24 prepubertal patients with sJIA, treated during at least the first 2 years (up to 7 years) with daily oral prednisone at a mean dosage of 0.2 mg/kg or more. Height data were expressed as the height standard deviation score for chronological age (HSDS/CA). A significant drop in height velocity (HV) (HSDS under − 2 SD) occurred during the first 4 years of the disease in 40% of patients and was correlated to the duration of prednisone therapy and with the severity of growth retardation during the active phase of the disease. Moreover, 70% of patients had a catch-up growth and 30% continued to show slow linear growth after prednisone discontinuation. Mean final height was below the target height in 80% of patients and was significantly correlated with mean height at prednisone discontinuation. These data suggest that early aggressive treatment increasing linear growth during the active phase of the disease could improve final height [4], and that aggressive control of inflammation, rather than GCs sparing, may be more important in the short-term to preserve normal growth [29]. On the other hand, the positive effect of steroid treatment needs to be balanced with the risk of long-term complications. In the last 10–15 years, immunomodulators and cytokines blockers have been introduced into routine clinical practice, managing to reduce the cumulative administered dose of GCs; furthermore, there is increasing use of intra-articular injections of GCs having less systemic side effects.

## Growth disorders in JIA and malnutrition

The term “rheumatoid cachexia” has been used to describe the condition of decreased lean body mass, reduced energy expenditure and increased catabolism which is often associated with inflammatory diseases, like JIA [30]. In humans, malnutrition is associated with hepatic GH resistance with elevated systemic GH levels. Short periods of fasting cause elevation of fibroblast growth factor-21 (FGF-21), which regulates gluconeogenesis, fatty acids oxidation and ketogenesis: FGF-21 inhibits GH receptor binding in growth plate chondrocytes through the induction of leptin receptor overlapping transcript (LEPROT) and leptin receptor overlapping transcript-like 1 (LEPROTL1) activity [31].

## Management of growth failure in JIA

Fighting the inflammatory process with immunomodulators (e.g., methotrexate), cytokine blockers (e.g., infliximab, etanercept, tocilizumab, anakinra), minimal effective use of GCs and optimal nutrition may improve growth, even if one-third of children with JIA continue to grow slowly and the reduction of disease activity does not seem to normalize linear growth in these patients [32].

Biologic treatments may reduce systemic inflammation and have a corticosteroid-sparing effect. Many studies have reported that anti-TNF treatments, the most widely used biologics, improve growth in patients with JIA [33]. Kearsley-Fleet L. et al. [34] evaluated the change over time in height z-score of 191 JIA patients treated with etanercept (ETN) for 2 years. Data about HV were compared with British median HV standards. The authors reported an improvement in mean height z-score during the first 2 years of therapy (from − 0.74 at the baseline to − 0.45), even if the cohort remained below the reference population mean, and patients with sJIA mantained the lowest z-scores (− 1.45). Mean HV was 5.8 cm/year, greater than that of age- and gender-matched peers. The factors most strongly associated with the growth improvement were a lower height z-score and no use of oral GCs at the baseline, even if patients receiving GCs at the start of ETN were mostly patients with sJIA. Moreover, data about patients’ pubertal stage were missing, and the study did not demonstrate a significant association between changes in disease activity (Juvenile Arthritis Disease Activity Score-71 JADAS-71 or Minimal Disease Activity MDA score) and improved height z-score. Another study reported a mean growth improvement of 0.45 SD after 2 years of therapy with anti-TNF-α in 51 out of 71 children with JIA, with no significant efficacy in patients with severe JIA [35]. A further report of 52 patients treated with ETN showed that pre-pubertal children had no significant height gain [36]. Uettwiller et al. compared growth velocity in 100 pre-pubertal children (Tanner Stage < 3) with JIA before and after the start of biologic therapy, continued for at least 6 months (mean duration of therapy 2.92 years) [32]. Patients were on follow up for 11 years on average. Fifty-one patients received anti-TNFα (ETN, infliximab, adalimumab), 12 were treated with the anti-IL-6 tocilizumab, 28 with anti-IL1β (canakinumab, anakinra), with the anti-CTLA-4 abatacept and 34 patients received more than one biologic drug. The authors did not show a significant difference between median HSDS at biologic treatment start and at the latest follow up, particularly in patients who received more than one biologic and in children with sJIA. In this study, the poor effect of biological therapy on growth could be due to the high proportion of patients with difficult-to-treat and sJIA. Nevertheless, 19% of patients remained below − 2 SD or under their target height at the latest follow up: these data describe a better growth compared with patients not receiving biologics, whose final height is below -2SD in 41% of cases in sJIA and in 11% of cases in polyarticular JIA [37]. It can be concluded that biologic treatment can improve growth in patients with JIA but it seems insufficient to restore normal growth velocity, especially in systemic and severe forms of the disease. Early initiation of biologic therapy and selecting the optimal first biologic agent could help improving final height in patients with JIA [32].

Given the alterations of GH-IGF-1 axis associated with chronic inflammation, recombinant human GH (rhGH) therapy, especially at high doses, could be a therapeutic option in pre-pubertal children with chronic diseases and in patients with pubertal delay who continue to grow slowly [10]. Moreover, in children with severe JIA, the prevalence of GH deficit may be up to 18% [38]. Various studies explored the role of therapy with rhGH in patients with JIA and growth delay. Earlier retrospective and prospective studies reported that therapy with rhGH in patients with JIA can improve HV, producing an adult height similar to the target genetic potential. They also suggested a better growth response with higher doses [39–41]. Simon D. et al. [42] studied the effect of 3 years of therapy with rhGH at high dose (0.46 mg/kg/week) given to 13 patients with JIA, evaluating the consequences on growth velocity, HSDS and body composition. They did not demonstrate significant changes of HSDS (from − 4.6 at baseline to − 4.3 at the latest follow up), but they showed an increase of lean mass by 33% and of lumbar bone mineral density (BMD) by 36.6%, preventing further bone loss. Fat mass remained stable and only 6 patients developed transient glucose intolerance, without developing diabetes mellitus. Other studies similarly showed that rhGH therapy in JIA children can decrease insulin sensitivity, so that close monitoring of patients by oral glucose tolerance testing (OGTT) is crucial before and during rhGH treatment, particularly during puberty and disease relapses [43]. More recently, a number of randomized control trials (RCT) assessed the efficacy of rhGH therapy in JIA patients [44, 45]. In a RCT of 31 children with JIA, patients treated with GH (0.33 mg/kg/week) for 4 years had an increase in height of 1 SD, compared to the decrease of 0.7 SD in untreated patients [46]. Long-term follow up data (7 years) indicate that mean final height was significantly greater in treated patients (SD -1.6 ± 0.25) compared to control group (SD -3.4 ± 2); besides, rhGH therapy did not affect age of puberty which was comparable between the two groups [38]. Simon D. et al. conducted an RCT study on a group of pre-pubertal children with recent JIA onset: after 3 years of therapy with rhGH (0.47 mg/kg/week) they showed a relative height gain of + 1.5 SD [47]. Small increase of lean mass, fat mass gain and lower bone mineral content (BMC) are often found in children with severe forms of JIA [48, 49]. Because of its anabolic effect, GH treatment, in these patients, causes reduction in fat mass and significant increase and normalization of total bone and muscle cross-sectional area (CSA) at final height [50].

There is now sufficient evidence to support that early use of relatively high dose of rhGH may improve linear growth in children with JIA. The acceleration in linear growth is associated with an increase of IGF-1 and IGFBP-3 serum levels, which persists during the continuation of therapy, showing a preserved GH-sensitivity [51]. However, catch up growth does not occur consistently and there is an important inter-individual variability in growth response [52]. The impact of therapy in patients with systemic and severe disease is still unclear. It seems that severe inflammation decreases the short- and long-term effect of rhGH therapy and that growth impairment during GH therapy correlates negatively with inflammatory markers and cumulative dose of GCs [53]. The role of pubertal stage should also be assessed, since the growth potential is also related to bone maturity: therefore, bone age should be considered, as a predictor for final HSDS.

## Puberty and JIA

Puberty is a critical transition life process in which a complex series of hormonal and neurological changes result in the physical development of sexually mature adults [54]. The mechanisms governing the timing of puberty are currently a rich field of research and they are not completely understood. However, it is clear that many exogenous and endogenous factors (nutrition, genes, inflammatory status, endocrine-disruptor agents or societal changes) can influence the pubertal neuroendocrine events [55]. The evident plasticity of puberty timing and the importance of environmental factors allows adaptive changes that represent at the same time a limit and a resource for adolescent growth and health [56]. The activation and the progression of puberty are strong surrogate markers of adolescents’ physical and mental health and this condition has obvious consequences in children with chronic diseases [56, 57]. Indeed, pubertal disorders in children with JIA, like in those with other inflammatory diseases, are frequently found, especially if they have prepubertal onset of disease and severe and prolonged chronic inflammatory course (sJIA) [58–60]. The most frequent pubertal abnormality described in these patients is delayed puberty, defined as the absence of testis enlargement or breast development at an age that is 2–2.5 SD later than the population mean (Tanner stage G2 in boys after the age of 14 years and B2 stage in girls after the age of 13 years in most Western countries) [61]. Slow clinical progression of puberty, isolated delayed menarche in females and decrease duration and intensity of puberty growth have also been reported [56]. However, few studies evaluating the prevalence of pubertal disorders, specifically in children with JIA, are available in the literature. Some authors found that the timing of menarche was later in all the 83 JIA patients in comparison with their mothers and normal Italian girls [58]. Similarly, other authors described a pubertal delay in 80 children with JIA compared to controls, less significant in the systemic onset JIA than in the oligoarticular and polyarticular groups [59]. In addition, other authors found a prevalence of delayed puberty of 15% in children with JIA, which is statistically significant compared to controls (1.4%), and an association between dose of corticosteroids, age at the administration of corticosteroids and the delayed puberty in boys [59]. Conversely, Ostensen M. et al. did not demonstrate significant differences between cases and controls in age of menarche, but they reported a reduced fecundity and increased incidence of gynecological disorders in 24 women with diagnosis of JIA in childhood [62]. Alike, other authors did not highlight differences in age of menarche and in adult height between 43 JIA girl and the 59 healthy controls [63].

Abnormalities in puberty may compromise the achievement of peak bone mass (PBM), lean mass and linear growth and may also lead to a psychosocial distress with lower quality of life in some of these adolescents. For these reasons, the assessment of pubertal status needs to be incorporated into the routine evaluation of adolescents with JIA [56, 64].

## Pathogenesis of pubertal disorders in JIA

Persistent inflammatory state, GC treatment and altered body composition with cachexia are mainly responsible for the alteration of regulation of GH and gonadotropins secretion through multiple mechanisms. Actually, each of these factors, may impact in parallel on two aspects of the pubertal process that mutually influence each other: the hypothalamic-pituitary-gonadal axis, on one hand, and skeletal development by effects on the bone and the growth plate locally, on the other [56].

## JIA and hypothalamic-pituitary-gonadal axis

The production of cytokines, in particular TNF-α, IL-1 and IL-6, has effects on the gonadotropins axis that are similar and concomitant to those described for those on GH-IGF-1 axis. The understanding of the mechanisms underlying the link between inflammatory cytokine-gonadotropin axis is given by few experimental studies of translational medicine. Inflammatory cytokines have been shown to act at the level of the central nervous system (CNS) [65]. IL-1-immunopositive fibers can be found in human and rat hypothalamus and IL-1 receptors are present in the rat brain. The injection of IL-1β in the cerebral ventricles of rats reduces the luteinizing hormone (LH) increase due to inhibition of LH-releasing hormone (LHRH) release [66]. This effect is mediated via IL-1-activation of hypothalamic endogenous opioid peptide system [67]. In rats not only intraventricular injection of IL-1 and TNF-α, but also of lipopolysaccharide (LPS) (which triggers secretion of cytokines) blocks the LH-LHRH axis [68]. This has been confirmed by similar studies, which demonstrated that LPS induces a profound transcriptional down-regulation of LHRH receptor gene expression in the anterior pituitary gland throughout the entire estrous rat cycle. While IL-1β appears the most potent inhibitor of the gonadotropin releasing hormone (GnRH)-LH system, IL-1α and TNF-α are less effective. The IL-6 role would seem marginal [69]. In addition, kisspeptin-RFamide-related peptide (RFRP) system is another link between inflammation and gonadal function [70]. In fact, kisspeptin-GPR54 signaling, particularly expressed in the hippocampal dentate gyrus, has an important role in initiating GnRH at puberty; instead, RFRP-3 has been implicated in GnRH neuronal inhibition in mammalians. It has been proposed that the balance between these systems is related to the maintenance of the fine-tune excitability of GnRH neurons and the timing of the preovulatory LH surge [71]. The injection of TNF-α in primary cultures of human fetal hypothalamic cells of GnRH neurons reduces GnRH secretion via downregulation of kisspeptin signaling [72]. On the other hand, LPS injection has been demonstrated to increase hypothalamic RFRP mRNA levels in rodents, thus increasing the negative GnRH- LH surge regulation mechanism [73].

The systemic administration of GCs, especially if prolonged and started in the pre-pubertal period, contributes to cause the GnRH-gonadotropin-gonadic axis alteration [74]. Such effects not only occur via centrally inhibition at the level of the hypothalamus and pituitary gland, but it is also the consequence of a reduced secretion of sex steroid from the gonads or impaired organ sensitivity to sex steroids [75]. Both chronic and acute administration of GCs in male rats result in alteration of reproductive axis: chronic use lead to a significant decrease of hypothalamic GnRH mRNA, while short-term GCs treatment is related to follicle stimulating hormone (FSH)-β mRNA levels decrease [74]. In addition, in vivo studies on adult women and adolescents treated with GCs affected by other rheumatological diseases highlighted a suppression of LH secretion and an upregulation of FSH levels, assuming the possibility of an effect on the gonadal function [76, 77].

## JIA and bone

The achievement of about 50% of the PBM is reached during puberty because of an increase in bone cortical thickness and a stimulus for trabecular mineralization, in parallel with an increase in growth plate enlargement [56, 78, 79]. Low PBM may result from clinical conditions associated with pubertal abnormalities or be directly a consequence of delayed puberty, highlighting the link between puberty and bone development [79–81].

**Fig. 2:**
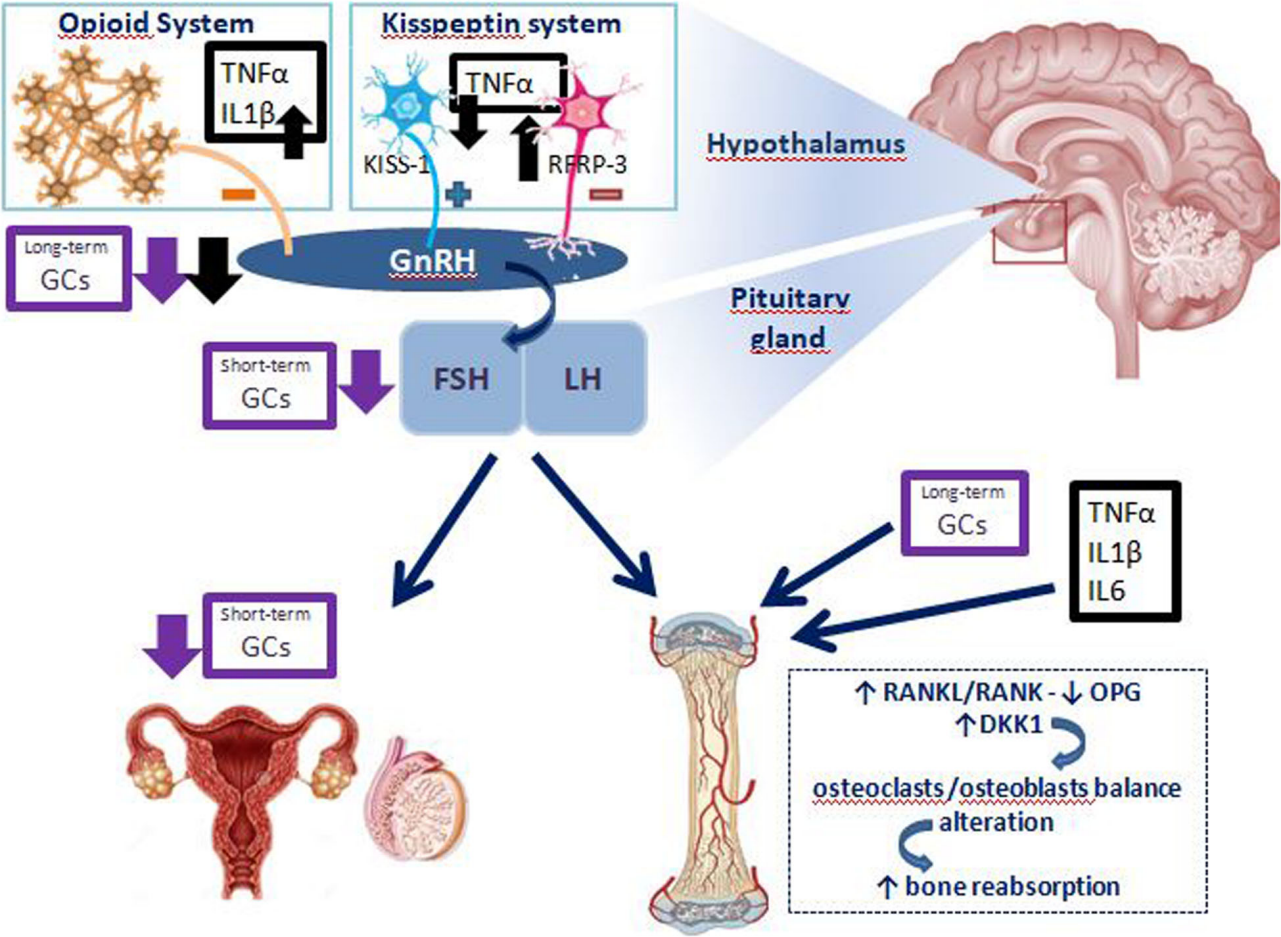
Effects of pro-inflammatory cytokines and of GCs on gonadotropin-gonads axis as well as on bone metabolism. IL-6 (Interleukin-6); IL-1β (Interleukin-1β); TNFα (Tumor Necrosis Factor-α); GCs (glucocorticoids); GnRH (gonadotropin releasing hormone); LH (luteinizing hormone); FSH (follicle stimulating hormone); RFRP-3 (RFamide-related peptide); Kiss-1 (kipeptin-1); OPG (osteoprotegerin); RANKL (nuclear factor kappa β ligand); DKK-1 (dickkopf-1)

The international study “The Bone Mineral Density in Childhood Study” demonstrated that the age at onset of puberty is a strong negative predictor of BMC and BMD in all DXA skeletal site in adulthood [82]. JIA patients with delayed puberty (34%) had significantly decreased BMD z-score values, compared to controls; in addition, the age of menarche seemed to be related to peripubertal mineralization [83]. In JIA children, many overlapping mechanisms are responsible for the uncoupling of bone formation from bone reabsorption [83–86]. The reduction of muscle mass, caused by malnutrition and physical immobility, reduces the force on bone load lines, which is an important stimulus for the deposition of bone matrix. Chronic GC administration may lead to a negative calcium balance, via the decrease of intestinal calcium absorption and the increase of renal tubular urinary calcium excretion [85, 86]. Pro-inflammatory cytokines (mainly TNF-α), the excess of GCs as well as the reduced gonadal hormonal stimulation act on the nuclear factor kappa β (NF-KB) ligand (RANKL)/RANK/osteoprotegerin (OPG) system and alter the balance between osteoclastic apoptosis and osteoblastic matrix formation. Moreover, some of the effects on peripheral bone reabsorption of the gonadal axis, mainly mediated by estradiol, seems to be mediated indirectly by IGF-1 [86]. Recently WNT signaling pathway and the WNT soluble antagonist dickkopf-1 (DKK1) appeared to be an additional mechanism. Secretion of DKK1 by the synovia is stimulated by inflammatory status and suppresses bone deposition acting on osteoblast differentiation [85]. Therefore, pubertal delay is an additional insult to the bone accrual children with JIA, in addition to inflammatory cytokines, GCs use, low physical activity and malnutrition, with the risk of osteoporosis [56]. The osteoporosis in children has been defined by “Official Positions of the International Society for Clinical Densitometry (ISCD)” published in 2013 as the presence of one or more vertebral fractures (VF) in the absence of local disease or high-energy trauma or Z-score of BMD or BMC ≤ − 2 (adjusted for size in cases of children measuring below the 3rd percentile) and a history of clinically significant fractures (two or more long bone fractures occurring by age of 10 years or three or more long bone fractures at any age up to age 19 years) [87]. As well as a reduction in BMD, evaluated by DXA, the presence of mostly asymptomatic VF has been found in more than 10% of JIA patients, especially with systemic and polyarticular onset [88]. This condition must be taken into account in the follow-up of children with JIA [89]. Fig. 2 illustrates the main effects of inflammation on GnRH-gonadotropin-gonadic axis and bone dysfunction.

## Management and therapy of pubertal disorders in JIA

Monitoring of puberty and bone health is essential for a complete health assessment of adolescents with JIA. In children with chronic inflammatory diseases such as JIA, an assessment of the pubertal stage through the Tanner scale every 6 months from the age of 9 years is recommended. In particular, the failure to initiate puberty over the age of 14 in boys and 13 years in girl should lead the pediatric rheumatologist to consult the pediatric endocrinologist in order to assess the need for induction of puberty [56]. Linear growth should be always evaluated at each medical examination: a pubertal delay, which can parallel growth retardation, can be recovered in many patients upon the disease remission [56, 79]. Evaluation of a wrist x-ray for bone age could reveal a constitutional delay in growth and puberty, which can be recovered once good disease control is achieved. Indeed, an appropriate disease control and the induction of clinical remission is the main goal to be achieved in children with JIA and pubertal disorder. Induction of puberty with sex steroid must be timely weighted together with the pediatric endocrinologist, identifying properly when the use of drugs acting on growth and the cytokine inflammatory activity are minimized. It is in order to allow adequate development of secondary sexual characteristics, evaluating potential for linear growth and the need to optimize bone mass accrual. In the medical literature there are no studies evaluating specific therapeutic sex steroid regimens for patients with JIA and pubertal delay. The use of standard therapeutic regimens offers multiple options such as intramuscular medroxyprogesterone acetate, transdermal 17-β-oestradiol patches or oral ethinyl estradiol or 17-β-oestradiol for girl and intramuscular testosterone enanthate orcypionate, propionate, transdermal testosterone 1% gel or oral testosterone undecanoate for boys [56].

Close monitoring of bone accrual is essential in JIA patients. Recently, the “Osteogenesis Imperfecta and Childhood Osteoporosis Working Group of the Spanish Society of Pediatric Rheumatology” published the guidelines for early diagnosis and treatment of osteporosis secondary to chronic conditions, including JIA [89]. Adequate nutritional vitamin D and calcium intake, exposure to sunlight on the hands, face and arms for6–8 min/day in the summer and 30 min/day during the coldest months of the year and adequate treatment of the disease represent the cornerstones for the optimization of bone health in JIA patients. A lumbar spine and total body less head (TBLH) DXA are recommended in JIA patients younger than 6 years in the presence of fragility fractures and in JIA patient older than6 years if not presenting rapid remission of disease in treatment with GCs. In patients treated with GCs a lateral spine x-ray is recommended at the beginning (within 6 months) of treatment and after 1 year, then every 9 to 12 months if treatment continues [89]. In patients who will receive GCs for 3 months or more, it is wise to give calcium supplementation or optimization of calcium (500–1000 mg/day) and vitamin D intake (400 IU/day), maintained for 3 months after discontinuation of GCs. Treatment with bisphosphonate (BP) is indicated in children with osteoporosis, according to ISCD 2013 position criteria, preferring the intravenous administration if a VF is present and oral treatment is in the decalage phase. The main important BPs used in children are shown in Table 1 [89, 90]. In addition, in early puberty (Tanner 2), the use of BPs presents additional indications, due to the importance of this phase in achieving the PMB. Indeed, BPs use can be considered, for pubertal patients with risk factors, or with BMD z-score ≤ − 2. 5 SD with a declining trajectory confirmed at least on two separate occasions with 1 year apart, or a BMDZ score ≤ −3DS with a declining trajectory confirmed on at least on two separate occasions with 1 year apart, even in the absence of osteoporosis [89].

**Table 1:** Main bisphosphonates used in pediatric age (adapted from Baroncelli G.I. et al.) [90]

Name	Oral	Parenteral	Dose	Relative potency
Etidronate	+	+	p.o.:5–40 mg/kg per day iv: 400 mg per day per 2 weeks, every 3 months	1
Clodronate	+	+	p.o.: 200 mg per day /subdivided into 3 doses iv: (dilute in 200–250 ml NSS, in 2–3 h) 2 mg/kg per day every 3–6 months	10
Pamidronate		+	(dilute in 100–250 ml of NSS, in 3–4 h)0.5–1.5 mg/kg per day for 3 days; every 2–6 months)	100
Neridronate		+	(dilute in 200–250 ml NSS, in 3 h)1–2 mg/kg per every 3–6 months	100
Zoledronate		+	(dilute in 50 ml NSS, in 30–45 min) 0.015–0.05 mg/kg every 3–6 months	> 10,000
Aledronate	+		1–2 mg/kg/week < 40 kg: 5 mg/day or 35 mg/week > 40 kg: 10 mg/day or 70 mg/week Maximum dose: 70 mg/week	100–1000
Risedronate	+		15 mg per week (< 40 kg) 30 mg per week (> 40 kg) Maximum dose: 30 mg/week	1000–10,000

*Abbreviations*: *iv* intravenous, *p.o*. per os, *NSS* normal saline solution

## Delayed puberty and growth impairment in patients with JIA: a close relationship

Delay of puberty in patients with chronic diseases may result in a decrease in duration and intensity of the pubertal growth spurt resulting in a loss of final height. Some authors reported that total pubertal growth (TBG) in 64 peri-pubertal JIA patients treated with GH (0.33 mg/kg/week) was increased by a factor of 1.5 greater in comparison to controls, with a gain of + 1.16 in height SD during puberty, leading to a significantly better final height [91]. In JIA patients treated with GH, mean TPG was 23.2 cm in boys and 19.8 cm in girls, lower than healthy children (20–35 cm), but significantly higher than controls (15 cm in boys and 12.5 cm in girls). Moreover, for each increase in age at puberty onset by 1 year, height gain was reduced 2.3 cm in GH treated patients and by 4.2 cm in controls during puberty, showing that GH could in part counteract the negative effect of pubertal delay on final height. This fits the suggestion to reduce the height deficit of short JIA patients before the onset of puberty. Some authors presented a long-term evidence (up to 10 years) that the adult height of sJIA patients can be normalized with an hormonal combination strategy, by postponing pubertal onset with a gonadotropin-releasing hormone analog until a more normal late-prepubertal height is reached or until the potential of prepubertal growth is exhausted and then promoting growth with high dose rhGH (0.5 mg/kg/week on average) when inflammation is reduced and high GCs doses are not needed [92]. In children with JIA, the reduction of GC doses and the initiation of a biological therapy favor an increment in height velocity, which does not evolve in a frank catch-up growth [93, 94]. On the contrary, if rhGH is added at high dose in a low-inflammatory condition, it is able to favor a prepubertal growth acceleration, comparable with the catch-up growth response in GH-deficient patients. This is also because this particular moment takes advantage of the period of relative glucocorticoid deficiency following prolonged steroid therapy, which is favorable to height gain [95].

## Conclusions

In patients with JIA, growth impairment and pubertal delay are well-known long-term complications. In the active phases of the disease, patients with polyarticular and sJIA show consistent growth impairment, while some children do experience “catch-up” growth following reduction in disease activity or reduction of GCs doses. Although new therapeutic options are available, there are still 10–20% of patients with severe forms of the disease who show continuous growth impairment, ending in a short final stature. Pubertal delay in patients with JIA can be a resource, as it can allow the adolescent to exploit the growth potential reserve, upon reaching the disease remission or the lowest disease activity. On the other hand, delayed puberty is associated with a reduction in the PBM with the possibility of concomitant or future bone fragility. Moreover, the effect of GH therapy is greater if started in patients with controlled disease, steroid-sparing therapy and pre-pubertal stage. Therefore, the clinician’s goal is to take advantage of patient’s linear growth potential and simultaneously optimize bone accrual and health. It is advisable that rhGH therapy should be a “target therapy”, tailored to the patient and based on pubertal stage, bone age, degree of systemic inflammation and ongoing treatment.

## Data Availability

Not applicable.

## Abbreviations

JIA: Juvenile idiopathic arthritis
ILAR: International League of Associations for Rheumatology Classification
sJIA: Systemic juvenile idiopathic arthritis
RF: Rheumatoid Factor
PsA: Psoriasic arthritis
GCs: Glucocorticoids
CAPS: Childhood arthritis prospective study
CHAQ: Childhood health assessment questionnaire
BMI: Body mass index
ReACCh-Out: Research in Arthritis in Canadian Children emphasizing Outcomes
GH: Growth hormone
IGFs: Insulin-like growth factors
GHRs: Growth hormone receptors
PI3K: Phosphatidyl-inositol-3-kinase
MAPK: Mitogen-activated protein kinase
IRSs: Insulin receptor substrates
PKC: Protein Kinase C
STAT: Signal transducers and activators of transcription
GHBP: Growth hormone binding protein
IGFBPs: Insulin-like growth factor binding proteins
ALS: Acid labile subunit
ILs: Interleukins
IFN: Interferon
IL-6: Interleukin-6
IL-1β: Interleukin-1β
TNF-α: Tumor necrosis factor-α
NSE: Neuro-specific enolase
JAK: Janus kinase
COMP: Cartilage oligomeric matrix protein
BAP: Bone alkaline phosphatase
MMP: Metalloproteinases
SOCS: Suppressor of cytokine signaling
HSDS/CA: Height standard deviation score for chronological age
HV: Height velocity
FGF-21: Fibroblast growth factor-21
LEPROT: Leptin receptor overlapping transcript
LEPROTL1: Leptin receptor overlapping transcript-like 1
ETN: Etanercept
JADAS-71: Juvenile arthritis disease activity score-71
MDA: Minimal disease activity
rhGH: Recombinant human growth hormone
BMD: Bone mineral density
OGTT: Oral glucose tolerance testing
RCT: Randomized control trials
CSA: Cross-sectional area
PBM: Peak bone mass
CNS: Central Nervous System
LH: Luteinizing hormone
LHRH: LH-releasing hormone
GnRH: Gonadotropin releasing hormone
FSH: Follicle stimulating hormone
LPS: Lipopolysaccharide
RFRP: RFamide-related Peptide
OPG: Osteoprotegerin
BMC: Bone mineral content
NF-KB: Nuclear factor kappa β
ISCD: International Society for Clinical Densitometry
VF: Vertebral fractures
TBLH: Total body less head
BP: Bisphosphonate
TPG: Total pubertal growth
